# Source Reconstruction Accuracy of MEG and EEG Bayesian Inversion Approaches

**DOI:** 10.1371/journal.pone.0051985

**Published:** 2012-12-21

**Authors:** Paolo Belardinelli, Erick Ortiz, Gareth Barnes, Uta Noppeney, Hubert Preissl

**Affiliations:** 1 MEG Center, University of Tübingen, Tübingen, Germany; 2 Wellcome Trust Centre for Neuroimaging, University College London, London, United Kingdom; 3 Cognitive Neuroimaging Group, Max Planck Institute for Biological Cybernetics, Tübingen, Germany; 4 Department of Obstetrics and Gynecology, University of Arkansas for Medical Sciences, Little Rock, Arkansas, United States of America; 5 Werner Reichardt Centre for Integrative Neuroscience (CIN), University of Tübingen, Tübingen, Germany; Université de Nantes, France

## Abstract

Electro- and magnetoencephalography allow for non-invasive investigation of human brain activation and corresponding networks with high temporal resolution. Still, no correct network detection is possible without reliable source localization. In this paper, we examine four different source localization schemes under a common Variational Bayesian framework. A Bayesian approach to the Minimum Norm Model (MNM), an Empirical Bayesian Beamformer (EBB) and two iterative Bayesian schemes (Automatic Relevance Determination (ARD) and Greedy Search (GS)) are quantitatively compared. While EBB and MNM each use a single empirical prior, ARD and GS employ a library of anatomical priors that define possible source configurations. The localization performance was investigated as a function of (i) the number of sources (one *vs*. two *vs*. three), (ii) the signal to noise ratio (SNR; 5 levels) and (iii) the temporal correlation of source time courses (for the cases of two or three sources). We also tested whether the use of additional bilateral priors specifying source covariance for ARD and GS algorithms improved performance. Our results show that MNM proves effective only with single source configurations. EBB shows a spatial accuracy of few millimeters with high SNRs and low correlation between sources. In contrast, ARD and GS are more robust to noise and less affected by temporal correlations between sources. However, the spatial accuracy of ARD and GS is generally limited to the order of one centimeter. We found that the use of correlated covariance priors made no difference to ARD/GS performance.

## Introduction

MEG and EEG are non-invasive neuroimaging methods that provide an exceptionally high temporal resolution. Moreover, MEG and EEG measurements stem directly from neuronal activation, whereas fMRI studies proxy epiphenomena, like blood oxygenation. However, the ideal approach for localization of neural generators of electrical/magnetic signals is still under debate [1]–[4].

Over the past decades, several algorithms have been developed for M/EEG source localization [4]–[9]. Since the inverse problem is ill-posed, prior information must be included to give a unique solution.

In recent years, Parametric Empirical Bayesian (PEB) approaches have been applied to MEG/EEG data for source reconstruction [10]–[15]. PEB theory imposes flexible constraints on the inverse solution in the form of source priors: for a given dataset, the most likely priors are those that maximize the model evidence.

Specifically, in a hierarchical linear model with two different levels, the parameters at first (source) level form an empirical prior for the second (sensor) level. The unknown covariances at each level are then expressed as a weighted linear combination of independent covariance components, one for each source prior. The contribution of each component to the general covariance is determined through its corresponding weight or hyperparameter.

In the PEB framework, the hyperparameters connected to the covariance components are iteratively adjusted based on the model evidence to select a set of brain sources which maximize the probability of the measured data.

From a Bayesian perspective, the simplest a priori assumption is the Minimum Norm Model (MNM). MNM estimates a source distribution that minimizes the error between the simulated field generated from the modeled sources and the observed neuromagnetic data, whilst simultaneously minimizing the overall source power [7]. This is translated in two practical assumptions: all the potential sources are a priori considered (1) equiprobable and (2) uncorrelated from each other (i.e. the source covariance is equal to the identity matrix) [10]. Recently, two algorithms based on a Variational Bayes (VB) approach with Laplace approximation [16] have been proposed, both distributed within the SPM software package (http://www.fil.ion.ucl.ac.uk/spm/): Automatic Relevance Determination (ARD) [10], [15] and Greedy Search (GS) [17]. ARD and GS covariance priors are based on a library of user-defined local spatial patterns (or patches), resting on the assumption that cortical currents exhibit some local coherence within a distance of few millimeters. The prior library is based on an arbitrary anatomical parcellation and does not depend on functional data. Additionally, no temporal constraint is imposed on the possible form of source activity. Both ARD and GS start their iterative selection process of the active priors with the assumption that all priors are equally likely to be active. The hyperparameters connected to the priors are then iteratively updated using a Restricted Maximum Likelihood (ReML) routine [18]. The so-called free energy F is the objective function of ReML, providing an approximation to the model evidence [16]. The iterative optimization procedure is different for the two approaches: ARD assumes a large number of putative sources and eliminates those that prove irrelevant for data explanation; GS starts from the assumption that all priors have identical variance and it tests putative mixtures of anatomical priors (rather than individual ones as in ARD).

In this work, we have implemented a new Bayesian scheme based on a Linear Constrained Minimum Variance (LCMV) beamformer [19] using a single covariance prior with strong temporal, but no spatial constraints. A unique solution for the inverse problem is obtained by imposing prior constraints derived from the sensor-data covariance [5]. Approaches based on beamformers [20] have been extensively utilized as tools for MEG/EEG source localization both in time and frequency domain [6], [19]–[31].

Beamformers are data-dependent spatial filters originally developed for radar technology [20]. The goal is to modify the sensitivity profile of a fixed array of sensors (like in the MEG and EEG cases) in order to get signals from a location of interest while signals coming from other locations are attenuated. Moreover, beamformers assume uncorrelated source time-courses. While some studies have shown that this assumption produces no evident bias with certain data sets [32], [33], other reported that it may induce severe biases when the level of correlation between sources and the signal to noise ratio (SNR) is high [34]. From the Bayesian perspective, beamforming can be considered an inverse scheme employing a unique prior: the beamforming estimate of source covariance. This prior depends on the sensor data covariance and the leadfields defining the source space. In contrast to the ARD and GS schemes, no anatomical parcellation is necessary but rather the prior constraints are temporal in that they minimize the covariance between sources. In the following, we compare the performance of different priors under the same ReML optimization framework. Each prior set defines a different algorithm: MNM, ARD, GS and an implementation of beamformer in a Bayesian framework, which we call Empirical Bayesian Beamformer, EBB [4], [35], [36].

Localization results with one, two and three sources and different levels of correlation between sources are evaluated. A new approach inspired by the free-response receiver operating characteristic (FROC) [37] method is employed to evaluate the spatial accuracy. Temporal accuracy is evaluated in terms of the amount of variance of the simulated source time courses explained by the reconstructed source time courses.

To summarize our findings, we found ARD and GS to be robust to noise, probably because of the iterative fine-tuning on the hyperparameters related to the source priors [10], [15]. On the other hand, the parcellation of the cortical surface imposes a trade off between spatial accuracy (improved by having more patches to give a denser coverage of the cortical surface) and robustness (the fewer patches, the less likely the algorithm is to get stuck in a local maxima). The VB algorithms were expected to perform better with bilateral correlated sources when the corresponding source priors were considered but we found no evidence for this. In contrast to ARD and GS, we found the performance of the EBB and MNM (which both use a source space with possible vertex precision and a single global prior) to be relatively poor except at high SNR.

The next sections are organized in the following way: we first outline the different stages of data analysis for the different schemes (2.1). Then, the preprocessing approach for the reduction of the data dimensionality is described (*2.2*). Forward and inverse models employed in data analysis are described in sections *2.3* and *2.4*. Then, an operative definition of Bayesian prior for the different schemes is provided in section *2.5*. The different priors used by the four schemes are described in detail in section *2.6*. A special focus on the mutual evaluation of the hyperparameters performed by ARD and GS is provided in *2.7*. The crucial differences between the two iterative approaches are outlined in *2.8*. The structure of the evaluation procedure for the four schemes (construction of simulations and accuracy estimation criteria) is described in *2.9* and *2.10*. Finally, the results are illustrated and discussed in the sections *3* and *4*.

## Methods

### 1 Stages of Data Analysis

**Figure 1:**
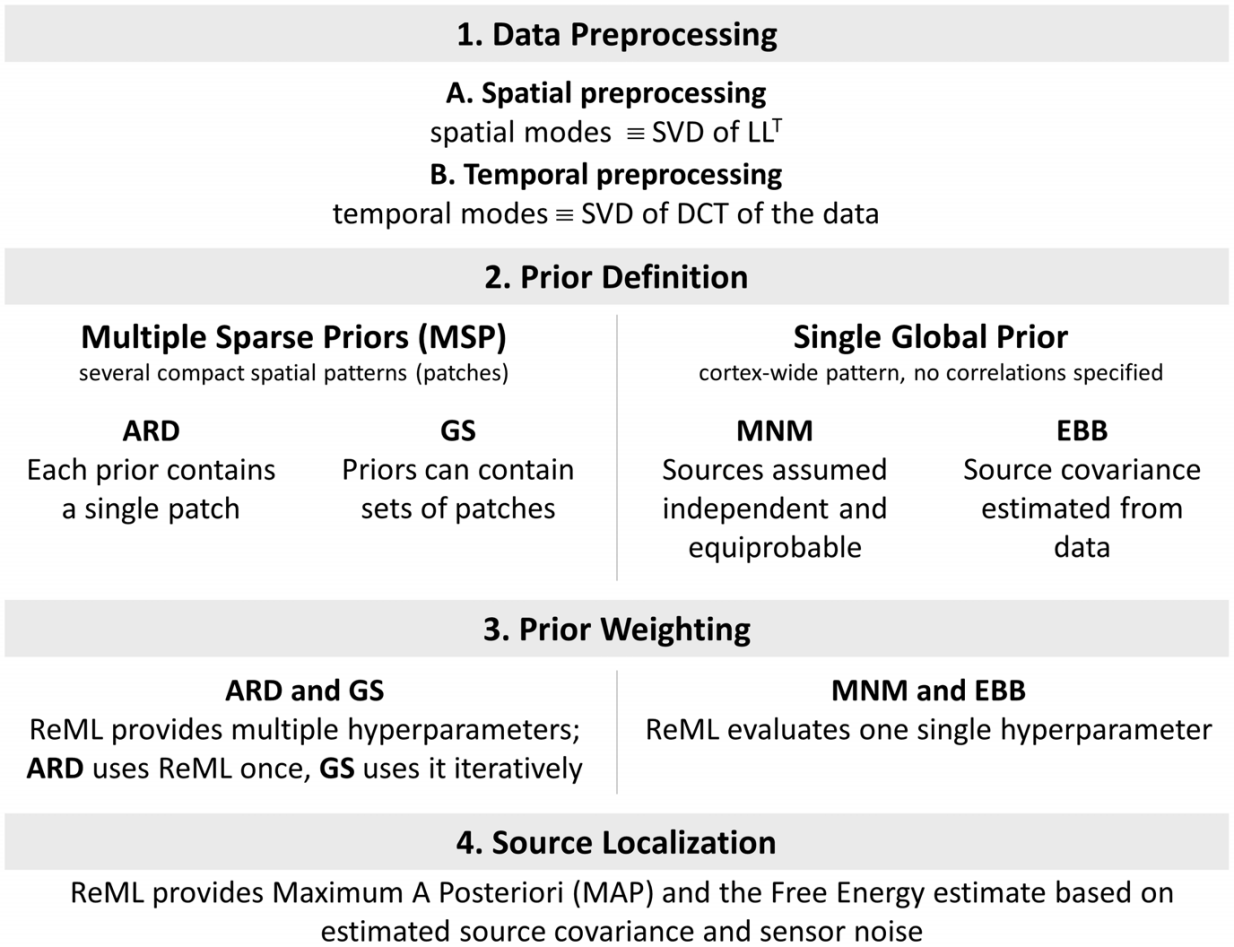
The four common stages for the algorithm comparison. 1. Data preprocessing (common to all schemes) 2. Prior definition (multiple (ARD and GS) or single (MNM AND EBB)) 3. Prior weighting through ReML 4. Source localization (again, common to the four schemes).

Our description of the different schemes will consist of four common stages (Fig. 1):

1. Preprocessing: this step is the same for all schemes. It includes (a) a *spatial preprocessing* selecting the dominant spatial modes based on the leadfields (the leadfield is the MEG/EEG signal that is generated by a source of unit strength); (b) a *temporal preprocessing* selecting the main temporal modes out of the data.
2. Prior definition: definition of a priori information to be used for the four inversion schemes.
3. Prior weighting: this stage implies the evaluation of the hyperparameters connected to the priors. This is done by means of a ReML procedure. In ARD and GS the relative weight given to the different priors will determine the localization results. In constrast, MNM and EBB rely on one single global prior over the source space. Therefore, no relative weighting is necessary.
4. Source activity extraction on the base of the three previous steps. A new ReML loop estimates the covariance matrix using the noise prior and the global source prior synthesized in the previous step. This step is independent of the scheme that generated the prior, whether EBB, MNM or MSP. Its output is used to calculate the maximum a posteriori estimate and provides a comparable value for the free energy of each scheme.

### 2 Data Preprocessing

All our analyses have been performed within the SPM framework.

Two steps must be performed before the application of an inversion scheme: (i) reduction of possibly rank-deficient data, (ii) explicit statement of prior expectations on unknown variables.

Our goal is to estimate activity and spatial location of electrical sources **S** from the measured magnetic data **B**:
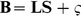
(1)Where 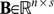 is the magnetic data matrix with *n* number of sensors and *s* number of time samples. The unknown sources are represented by 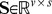 where *v* is the number of points on the tessellated surface, which are possible sites for the active dipoles. 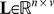 is the leadfield matrix, **ς** represents the sensor error due to noise and interference.

The dimensions of the above problem can be reduced by projecting the data into spatial and temporal subspaces. A spatial projector **U** and a temporal projector **T** determine the subspaces whose dimensions are spanned by the eigenvectors of the leadfields and the data, respectively [12]. The spatial projector **U** depends exclusively on the forward model. It is obtained by means of a Singular Value Decomposition (SVD) of the **LL** ^T^ matrix. The eigenvectors are ordered by their eigenvalues and arranged in columns of **U**, called *spatial modes*. The default selection removes all modes with a **LL** ^T^ eigenvalue inferior to e^−16^ of the mean. Then, a new matrix 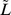 with *n* spatial modes (typically between 60 and 80) is considered instead of the original leadfield matrix **L** containing 275 channels. The temporal dimension of the data is reduced in a similar way. In this case a projection matrix **T** follows the application of the spatial projector **U**. Firstly, the spatially reduced data is DCT (Discrete Cosine Transform) transformed into the frequency domain. Any desired windowing or frequency filtering is applied at this stage. Then, as with the leadfields, the DCT coefficients are multiplied by their transpose and an SVD is used to identify the number of dominant temporal modes. Finally, by applying the inverse DCT to the reduced eigenvector set, we obtain a subspace spanned by a set of eigenvectors named *temporal modes*.

In summary, each element 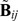 belongs to the spatially (*i*) and temporally (*j*) reduced signals 
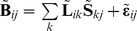
, that are our modeled signals in the reduced space.

Note that the data projection in temporal and spatial modes has another function besides the efficient utilization of computer resources: it also removes noise, allowing the procedure to focus on the effects we intend to explain. However, as with any data reduction, there is a risk of data loss, especially under very low SNRs.

### 3 Forward Model

For the source space, a tessellated surface of the grey-white matter interface with 8196 vertices is employed [38]. Each vertex corresponds to a possible source location. The source orientations are fixed, perpendicular to the surface. The mean distance between neighboring vertices is 5 mm. The leadfields are calculated using a single-sphere volume conductor model. The head, sensor positions and orientations are based on a real recording from a CTF 275 whole head system (VSM Medtech, Port Coquitlam, Canada).

### 4 Inverse Problem

The inverse problem can be treated with a hierarchical linear model on the reduced data. In this way (1) reduces to:
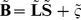
(2)

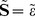
(3)


Where 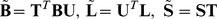


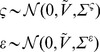
(4)

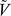
 denotes the temporal correlations in the reduced space which are assumed fixed and stable. As in [10], the three-parameter notation for a multivariate normal distribution is defined as 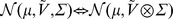, where 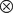 is the Kronecker tensor product. This preprocessing procedure is common to all the inversion schemes considered here.

### 5 An Operative Definition of Prior

From a Bayesian perspective, a *prior* is a probability distribution that expresses the uncertainty about an unknown variable before the data is taken into account [10]. ARD and GS are defined as Multiple Sparse Priors (MSP) schemes. In this case, the priors are source covariance components. At the simplest level, the single component is local with an extent of a few millimeters across the cortex (*sparse local prior*).

The source level covariance components can be compactly expressed in terms of sensor covariance components. Priors employed to estimate the sensor covariance matrix generated by the active sources, are defined as *covariance priors*. In this way, a covariance prior at sensor level is obtained for each local prior at source level. Since the estimated covariance is calculated as a combination of these priors, we refer to them as *covariance components*.

In contrast to the MSP schemes, MNM and EBB use a single, *global functional-anatomical prior* (functional because it is based on assumptions about source covariance and anatomical because it is constrained to the cortical manifold) provides just one estimated covariance component at sensor level.

### 6 Choice of the Prior Set

#### 6.1 Minimum Norm (MNM)

All the sources are assumed equiprobable and uncorrelated. Therefore, the source covariance matrix is defined as **Q =** ***I***. Only one hyperparameter is estimated by ReML on inversion step #3 (See Fig. 1).

#### 6.2 Empirical Bayesian Beamformer (EBB)

EBB assumes one global prior for the source covariance main diagonal (the off-diagonal elements are zeros, i.e. no correlations assumed). The Empirical Bayes differs from the traditional Bayes in that the priors are estimated from the data. Indeed both GS and ARD algorithms are empirical Bayes formulations, as well [39]. For every site *θ* the source variance is calculated in the following way [21], [40]:
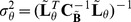
(5)


Where 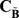 is the reduced data covariance and 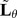 denotes the reduced leadfield. If we define the vector 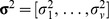 as the ordered set of source variances, we can then write the EBB covariance prior as:
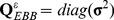
(6)


As in the case of MNM, ReML estimates only one hyperparameter in the EBB scheme.

#### 6.3 Multiple sparse priors (ARD and GS)

**Figure 2:**
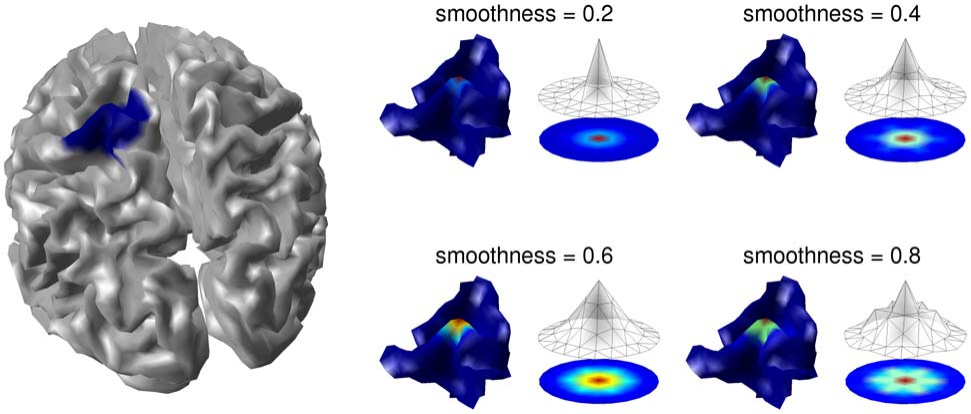
Profile intensities G(*s*) of the same spatial pattern for different values of the smoothness parameter *s.* LEFT: the location and maximum extent of the spatial pattern is shown (blue region). RIGHT: the spatial pattern is shown for different *s* values ranging from 0.2 to 0.8, projected on the cortical surface (left) and on a flattened surface upon a wireframe height map (right). The number of points featuring more than 60% of the G peak value (central point of the spatial pattern) can range from 20 (*s* = 0.2) to 55 (*s* = 0.8).

ARD and GS employ multiple empirical priors that are data independent but locally determined on the basis of brain anatomy. The generic source prior 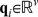 is a distributed pattern with compact spatial support. The spatial extent of a source prior is determined by a smoothing operator that employs the Green function:
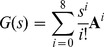
(7)where the generic element 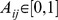 of **A** denotes the neighborhood properties of the vertices. Depending on the smoothness parameter *s*, the G function connects the patch points from a central vertex up to its 8th-order neighbor. Fig. 2 shows how different smoothness values affect the form and extent of G. In SPM, a trade-off value between spatial accuracy and local coherence is assumed by choosing *s* = 0.6. This choice provides an effective local coherence of approximately 10 mm.

The covariance component related to the single patch can be formulated in the following way: 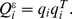 The minimum number of covariance components considered in this paper is 2*p* under the assumption of uncorrelated patch activities, where *p* is the number of patches per hemisphere (in this work *p* = 256). As a consequence, the estimated covariance can be expressed as the sum of the single patch covariance components weighted by their respective hyperparameters 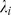 through the scale parameters 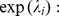.
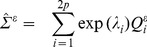
(8)


Theoretically, an infinite number of source priors could be generated to accommodate any linear combination of components. In our simulations, in addition to the minimal set of 2*p* components, we consider *p* elements of the following kind: 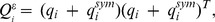 where 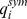 is the contralateral replication of *q_i_*. The inclusion of this prior set accommodates correlations between symmetrical areas of the two hemispheres. To test whether the addition of symmetrical correlated priors is beneficial, we performed every simulation set either with or without this set of components (i.e. with 3*p* or 2*p* priors, respectively).

### 7 Hyperparameter Tuning

The four schemes employ ReML (see Appendix S1 for a description) to estimate the hyperparameter set that determines the weight of each covariance component. Since in the case of MNM and EBB only one prior is considered, the ReML output is a single hyperparameter (i.e., a rescaling factor for the unique covariance component). In contrast, in ARD and GS, the N_C_ hyperparameters are iteratively evaluated at each ReML cycle.

The estimated log-evidence of the reduced data 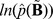 is the objective function. In fact, rather than maximizing the estimated evidence 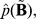 it is more convenient to consider the log of the same quantity in the following form:
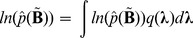
(9)


Where 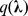 is the approximation of the conditional distribution 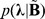 for the set of hyperparameters 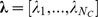 and *N_C_* is the number of covariance components. Under the Laplace approximation, the estimated conditional density of the hyperparameters is a Gaussian distribution 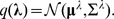 Mean and variance of the hyperparameter distribution 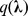 are estimated with a second-order Fisher scoring procedure [16] by means of the M-step of ReML (see Appendix S1).

Since the measure of the discrepancy between the conditional density and its approximation 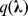 (also called Kullback-Leibler (KL) divergence).
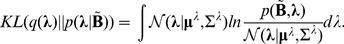
(10)is a positive quantity, the free energy F denotes a lower-bound for the log-evidence:



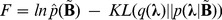
(11)The goal of ARD and GS is to get an approximation of the data log-evidence. By approximating 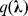 to 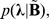 the KL divergence is minimized and F becomes a satisfactory approximation: 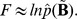


Unfortunately, the free energy F in (11) cannot be computed in closed form. Therefore, an approximation is used, giving a Gaussian prior density on the hyperparameters 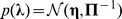:
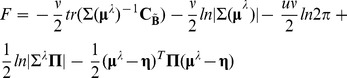
(12)
*u* and ν are the numbers of the reduced spatial and temporal dimensions. 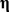 is a vector of N_C_ elements with the same mean value η. The covariance of the prior distribution 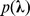 is a diagonal matrix: 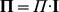. A Gaussian distribution assumption on **λ** is equivalent to assuming a log-normal distribution on the scale parameters 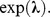 In the SPM framework, the values for η and Π are user-defined. We used the default values η = −32 and Π = 1/256 in this work. These values implement weakly informative (flat) priors providing a small expectation and a very large variance [41]. A variance of 256 for each hyperparameter *λ_i_* means that the scale parameters exp(*λ_i_*) is allowed to vary by several orders of magnitude. Assuming η = −32 implies that the expected mean value for all the scale parameters is around zero at the beginning of the ReML process.

The first term in (12) denotes the estimation accuracy (similarity between the estimated covariance and the reduced signal covariance). The second term is a measure of the estimated signal amplitudes which needs to be minimized. The third term is constant and depending on spatial and temporal dimensions of the reduced signal space. The last two terms quantify the complexity. They represent the similarity between the prior and posterior statistical moments of the hyperparameter distributions.

The MSP schemes focus on the estimation of source covariance 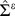 defined in (8) as a linear combination of several independent components 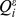 weighted by their respective scale parameters. The sensor noise covariance can be regarded as a single component that is linearly added to the signal components:
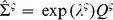
(13)where 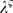 is the noise hyperparameter. The independence assumption over channels implies 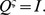 If we project the estimated source covariance 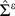 into the sensor level, the signal covariance can be expressed as a linear combination of signal and noise components:



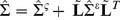
(14)In this way, the component estimation of 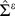 takes place at sensor level. Second and first level hierarchies of our model are collapsed into a single level. Basically, each scheme for source reconstruction can be considered as a tool for estimating the set of covariance components 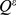 [4], [42].

### 8 Iterative Learning in ARD and GS

#### 8.1 ARD

**Figure 3:**
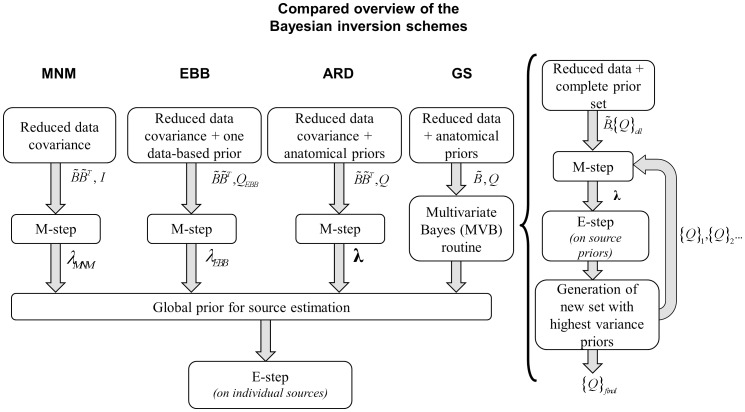
Overview of the four schemes pipeline. In the MNM and EBB case the M-step just provides a scaling factor on its single prior, while ARD uses it to weight and select source priors which give a relevant contribution. GS handles proposed sets of priors, discarding the irrelevant ones (applying the M-step), and introducing a new set with the most active priors (as estimated by the internal E-step). Finally, the common E-step at the end is the only stage where individual sources are evaluated.

ARD is a relevance determination scheme which operates solely on data covariance 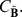 The estimated source covariance projected into sensor space is 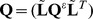. The ReML-step iteratively estimates first and second moment of the hyperparameters (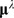 and 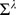) until convergence. As the conditional mode of the scale parameter connected to the i-th patch 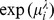 approaches zero (i.e. 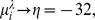 the hyperparameter reaches its prior expectation) at some point of the iterative process, so does the connected variance component 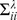 (gaussian assumption on 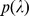). In this way, the i-th patch is discarded from the active set of patches. Upon convergence on the optimal hyperparameter set, the maximum a posteriori **M** matrix is calculated by means of an E-step only once. Convergence is reached when F stops increasing or only one active patch is left (Fig. 3).

#### 8.2 GS

In contrast to ARD, GS evaluates sets of patches rather than single elements. However, in the patch selection for each set, the relative weight of every patch within the set needs to be evaluated. Thus, at each ReML step, patch activity estimates are performed employing the quantites 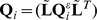 and 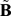 in one E-step in the reduced sensor space. That is, GS makes use of both the original data and the covariance components (Fig. 3).

GS works iteratively in two steps:

Step one:

Each set of patches has one covariance component with an associated hyperparameter. The hyperparameter evaluation process is implemented by ReML through an iterated M-step. The starting prior set for the first M-step uses all the components with the same variance. At each subsequent cycle, a new set is created which is a subset of the last one.

Step two:

The source level activity due to all prior covariance components is evaluated through an E-step (see Appendix S1). The individual source priors are then ordered according to their magnitude and the top half of the set is used to form a new, prior set. In this sense, the new set is a sort of genetic crossover which is likely to discard some of the parent sets in the next ReML iteration. This pruning keeps the number of current sets small (usually between 3 and 8).

The search terminates when the free-energy stops increasing or when the number of prior components reduces to one. Since each new set is smaller (by a factor of two) than the previous one, the search is extremely fast.

### 9 Construction of Simulations

Source localizations were performed on simulated datasets with one, two and three dipoles. In EBB and MMN it is possible to use all mesh vertices as possible source locations (as they are based on single dipole models). In contrast, ARD and GS are based on cortical patch models consisting of many dipoles, and these patches are relatively few in number (256 per hemisphere). In order to perform an appropriate comparison between the two solution spaces, all simulated dipole locations in the study were at patch centers.

**Figure 4:**
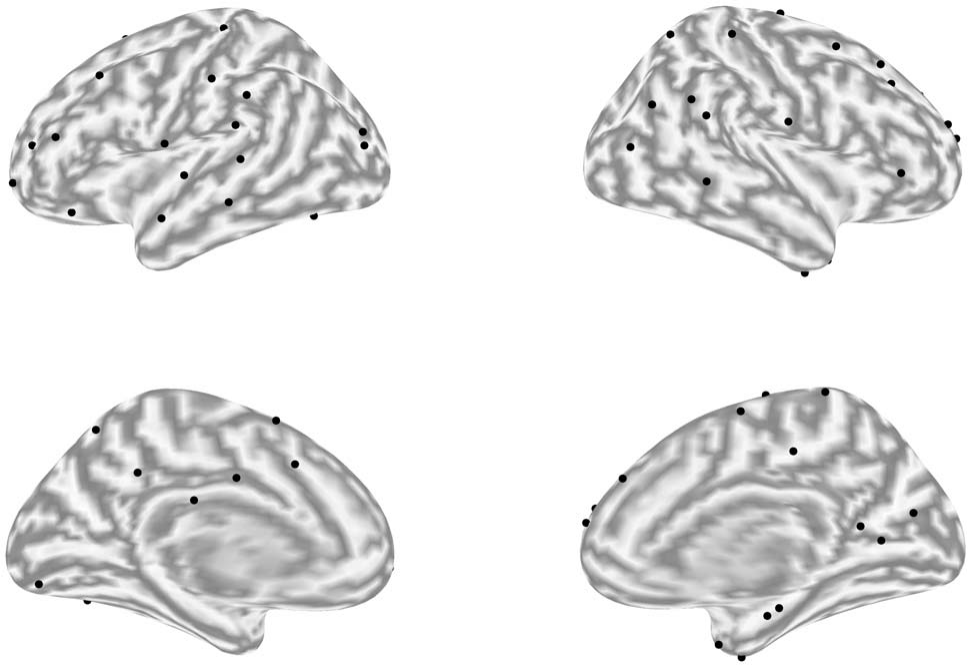
Possible simulated source locations. External and internal views of the brain hemispheres are shown in the upper and lower part of the image respectively. The algorithm for the source site choice aims to minimize the spatial pattern overlap.

A set of 50 single dipoles was selected out of 10000 random sets by selecting those with minimally correlated leadfields. While this does not necessarily guarantee a minimum mutual distance between the 50 locations, it achieves a satisfactory distribution across the source space. The final set is shown in Fig. 4. For the two-dipole simulations, we added either a dipole at the contralateral location (symmetrical configuration), or at a random one (asymmetrical) to evaluate the effect of bilateral correlated priors on ARD and GS performance. For the three-dipole simulations, the locations were also selected randomly.

**Figure 5:**
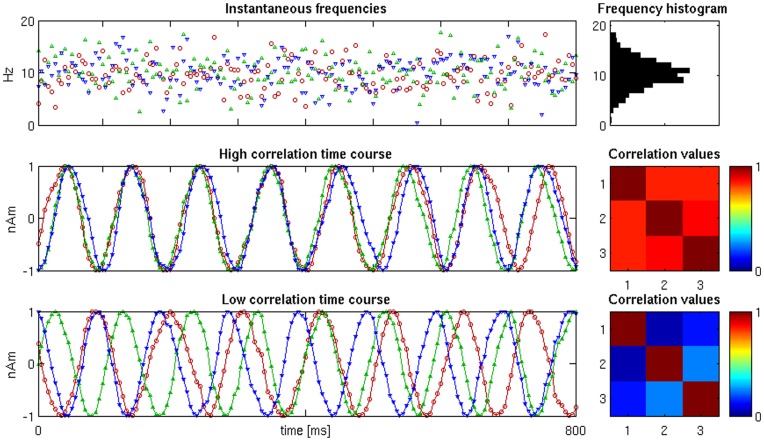
Synthetic time courses for one simulation. For each source, a frequency per time sample is drawn from a Gaussian distribution 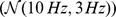, top row). The instantaneous source amplitude is obtained by integrating the frequencies and taking the sine of the resulting angle. If the generated time course satisfies the desired correlation threshold (either high or low: middle and lower rows, respectively), it is accepted; otherwise the procedure is repeated. The corresponding frequency histogram and correlation matrices are shown in the right column.

**Figure 6:**
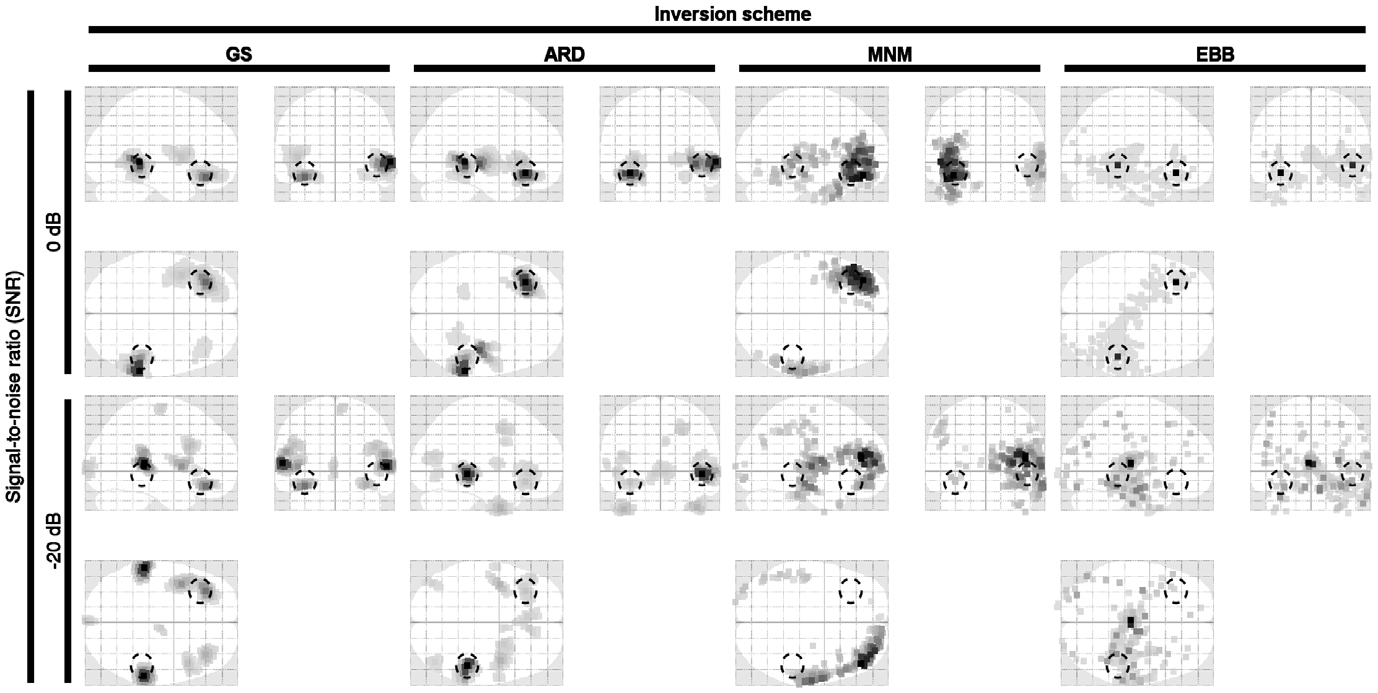
Example of localization performances at 0 and 20 dB. Two asymmetrical, weakly correlated sources are simulated in the forward problem. Symmetrical correlated priors are considered for ARD and GS. The actual simulated dipoles are centered at the dashed circles. EBB performs almost flawlessly at high SNR at high SNR. GS and ARD still show some local maxima in the actual source location at extremely low SNR.

Each simulation comprised 100 epochs of 0.8 seconds and a sampling rate of 200 *Hz*. One time course per dipole was generated for each simulation. For each time sample, an instantaneous frequency was drawn from the Gaussian distribution 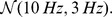 The time course was obtained taking the sine of the cumulative sum of the instantaneous frequencies, plus a random starting phase (Fig. 5). For the multiple-dipole simulations, the dipole time courses were controlled either for high (>0.8) or low (<0.3) correlation. Finally, these time courses were replicated over all trials. Each time-course had time-varying noise added to reach an SNR in the range of −30 to 10 dB, with steps of 10 dB. The SNR levels were set up by adding Gaussian noise to the sensor level data. The signal was defined as the average root-mean-square value of the noiseless sensor readings. Therefore, each simulation consisted of a dataset with 100 trials based on the same source locations and time-courses with the addition of random noise (varying from trial to trial). The four inversion methods were applied to give four image volumes for each trial. These volumetric current estimates were quantified in terms of spatial and temporal accuracy. In Fig. 6 an example of source localization is shown with noise levels at 0 and −20 dB for asymmetrical uncorrelated sources. For ARD and GS, symmetrical correlated sources were included in the set of source priors.

At 0 dB, ARD, GS and EBB demonstrate a satisfactory localization performance. MNM detects the lower source slightly misplaced towards the brain surface. At −20 dB, EBB does not localize the sources distinctly whereas both ARD and GS can localize one source in the right hemisphere and find a local maximum at the location of the left hemisphere source. In this case, MNM does not perform as well as the other algorithms.

### 10 Accuracy Parameters

#### 10.1 Spatial Accuracy Index (SAI)

**Figure 7:**
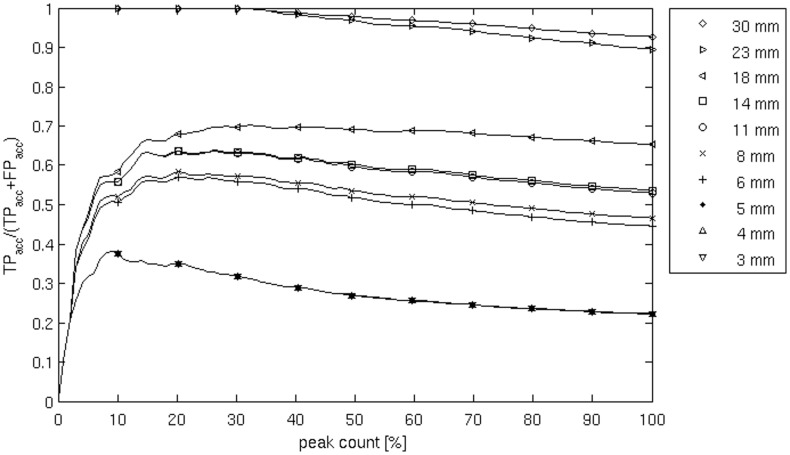
Example of a Positive Predictive Value (PPV) curve for one simulation’s source localization, calculated by means of the localization image volume. The PPV is the proportion of the images peaks that are localized within a given search-size around the simulated dipole (True Positives, TP). Peaks localized outside of the search-size are considered False Positives (FP). Thus, PPV is TP/(TP+FP). The peaks are ordered by intensity, and PPV is calculated for each fraction of the total peak count. In this way, a curve is obtained that indicates whether the stronger peaks fall near the dipole (decreasing slope) or far from it (increasing slope). The area under the curve was used as a performance indicator, the Spatial Accuracy Index (SAI). The curves in the figure depict 10 search-sizes from 3 to 30 mm, on a logarithmic scale.

To evaluate spatial accuracy, we used an approach inspired by the FROC methodology [37], [43]. FROC is an evaluation method that measures the overlap between simulated extended sources and detected ones. In contrast, our method evaluates the performance by measuring the distance between the local maxima of the estimated activity and the actual simulated dipole positions. As a first step, the brain volume is scanned to get a list of local image maxima. Peaks with values below 5% of the maximum peak have been removed to avoid noisy local maxima biasing the results (i.e. only the top 95% of peaks were considered). We count True Positives (TP) as the number of local maxima that fall within a distance *r* (our *search-size*) of one of the simulated dipole sites. We considered search-sizes ranging from 3 to 30 mm on a logarithmic scale. The local maxima detected more distant than *r* from a dipole are labeled as False Positives (FP). Then, the peaks are ordered by descending magnitude. Accumulated magnitudes TP_acc_ for TP and FP_acc_ for FP are calculated. Finally, a curve of the magnitude ratios Y = TP_acc_/(TP_acc_+FP_acc_) is computed. The area under the curve (AUC) can be taken as a performance index for the chosen search-size. We define this as as the *Spatial Accuracy Index* (SAI) ranging from 0 (no TP) to 1 (no FP, ideal case). In contrast to typical ROC curves our function is not necessarily monotonic (false positive detection, mostly when true positives have already been detected, lowers the ordinate value Y (Fig. 7)).

In table 1 the SAI values of the localization example in Fig. 4 are reported as example.

**Table 1:** SAI results for the four inversion schemes in the simulation trial presented in Fig. 4.

	GS	ARD	MNM	EBB
**SAI at 0 dB**	0.09	0.76	0.20	1.00
**SAI at −20 dB**	0.05	0.66	0.01	0.00

#### 10.2 Temporal Accuracy Index (TAI)

**Figure 8:**
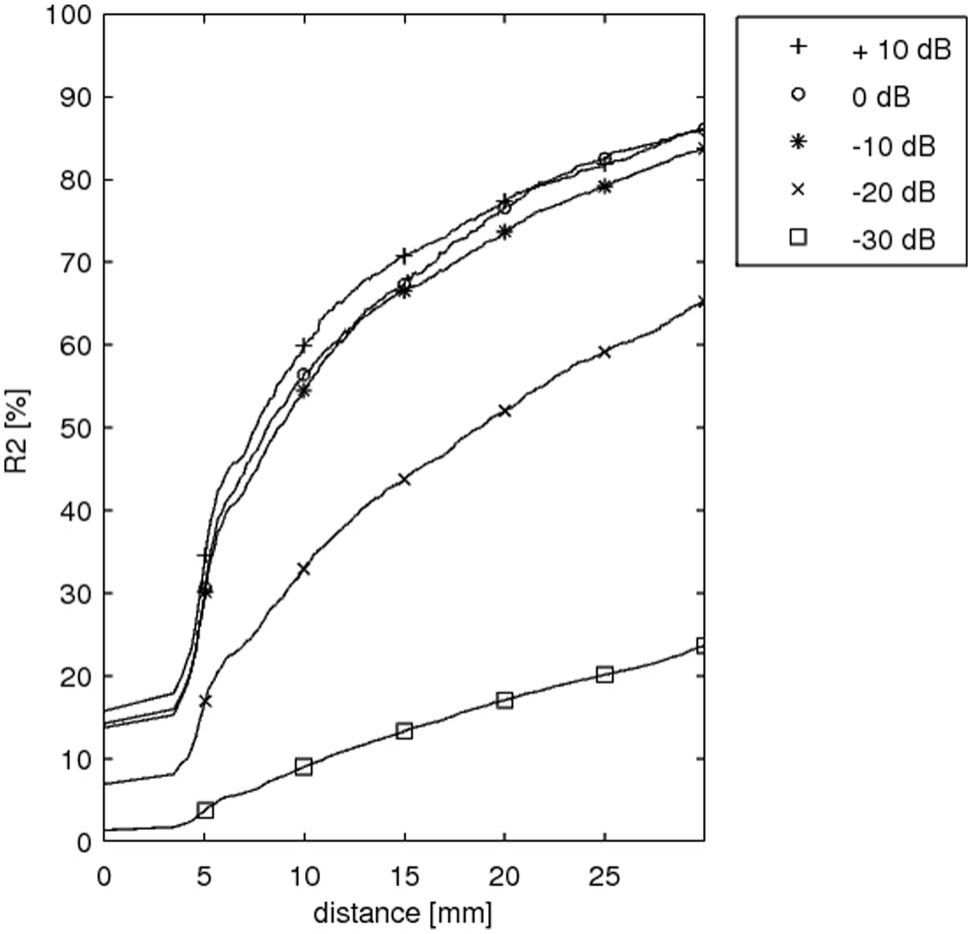
Example of curves of temporal variance explained by the source reconstruction (R^2^). The R^2^ value reported by SPM includes all vertices in the cortical mesh. Only the vertices located within the given search-size (x axis; 3 to 30 mm) were considered to generate time-courses. One line plot is calculated for each SNR (−30 to +10 dB, in 10 dB steps). The Temporal Accuracy Index (TAI) for a given search-size is considered as the R^2^ value at that distance.

In order to evaluate the *Temporal Accuracy Index* (TAI) of the reconstructed source time series we calculated the percentage of data variance explained by those sources labeled (in the previous step and depending on search size) as true positives. The explained data variance was quantified by means of the coefficient of determination
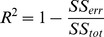
where
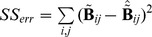
is the residual sum of squares. 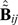 is the estimated field component generated by the reconstructed sources.



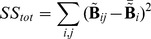
is the total sum of squares (proportional to the sample variance). The plotted curves of R^2^ are monotonic with respect to the search size because this quantity is bound to increase as the number of sources used to explain the variance is increased. We define Temporal Accuracy Index (TAI) as the area under the curve (Fig. 8). In the single dipole case we found that the dimension reduction in the preprocessing stage effectively removes all noise, with the exception of the lowest SNRs. As a general consequence, this implies that only one reduced time sample is present in such circumstance. Hence, R^2^ (and therefore TAI) is not defined for the single-sample case.

## Results

**Figure 9:**
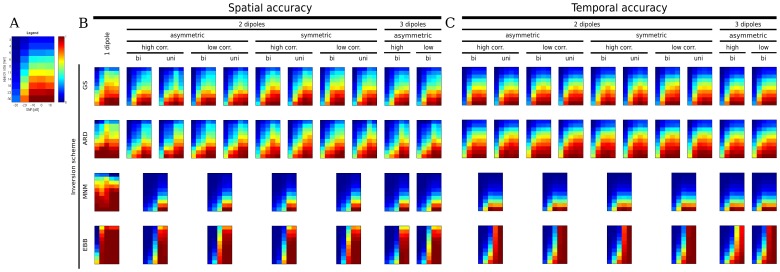
Summary of spatial (SAI) and temporal (TAI) accuracies of the four algorithms. A: Magnified example of a scale value grid for explanatory purposes. The color coded values represent the areas under the curve (AUC) pertaining to the spatial and temporal accuracy curves. AUC values are plotted as functions of SNR (*x* axis, −30 dB to 10 dB) and search-size (*y* axis, 3 mm to 30 mm, downward direction, logarithmic scale). B, C: Spatial (B) and temporal (C) accuracies were evaluated for 1, 2 and 3 dipoles. Different conditions were manipulated: (1) Symmetry of 2 sources (symmetric *vs.* asymmetric in the two hemispheres); (2) Correlation level between sources (high or low, for 2 and 3 sources); (3) Bilateral correlated source priors *vs* absence of them (only ARD and GS). ‘bi’ stands for correlated priors included. ‘uni’ stands for correlated priors omitted.

Fig. 9 shows the summary of results as a color map for spatial and temporal accuracy indices. Each color matrix shows the AUC (SAI or TAI) results for one method at a given source configuration. Each configuration is defined by (1) number of dipoles (one, two, three), (2) dipole locations (asymmetric, symmetric), (3) correlation between dipole time-courses (low, high) and (4) priors included in the source localization (*bi* = bilaterally correlated, symmetrical priors added to the single source priors; *uni* = only unilateral source priors included). In each matrix, the row and column indicate the search-size and SNR, respectively. The search-size ranges from 3 to 30 mm in a log scale, and the signal to noise ratio (SNR = 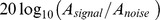) grows linearly from −30 dB to +10 dB. In general, GS and ARD have similar performances, across all conditions. When the acceptable localization error (or search-size) decreases, so do the accuracy measures. In contrast, EBB has close to perfect performance at higher SNRs but degrades relatively quickly for lower SNRs.

MNM has a good performance when applied to one dipole configurations. However performance degrades rapidly as the source configuration becomes more complex. Note that there is no discernible difference when bilateral priors were used in ARD/GS rather than just unilateral ones. These findings are presented in more detail in the next section.

### 1 SAI Results

**Figure 10:**
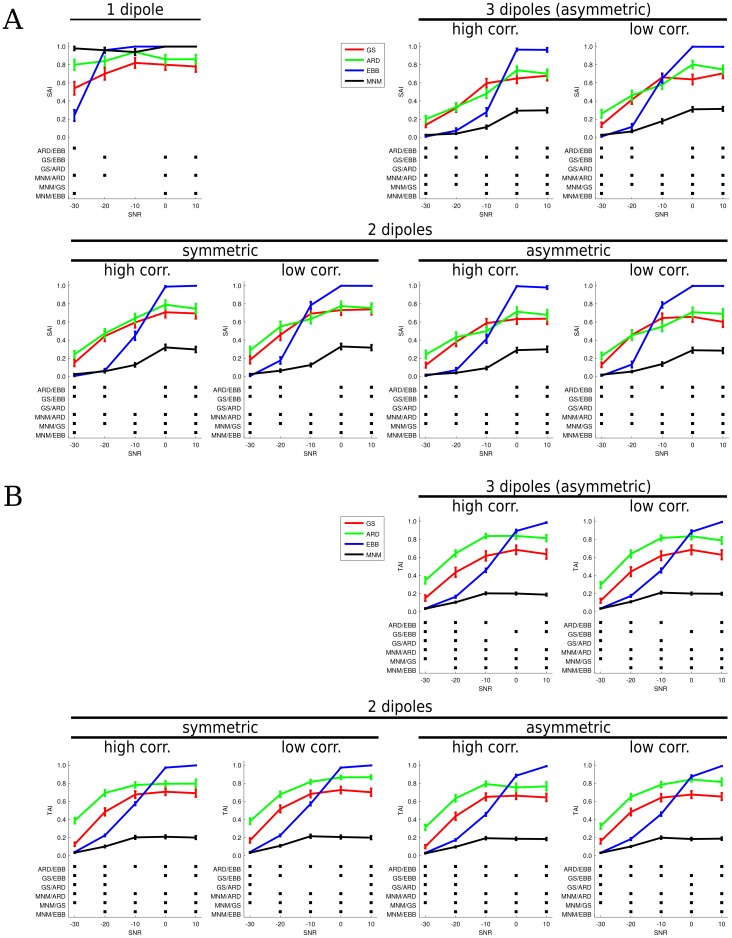
Statistical comparison of the inversion schemes for a search-size of 14 mm. ARD (green), GS (red), EBB (blue) and MNM results are plotted in Panel A (SAI results) and B (TAI results). For each simulation the mean accuracy index is plotted versus the different SNR levels. The error bars show the standard error. Black squares in the lower panels indicate significant difference between the schemes’ performances.

**Figure 11:**
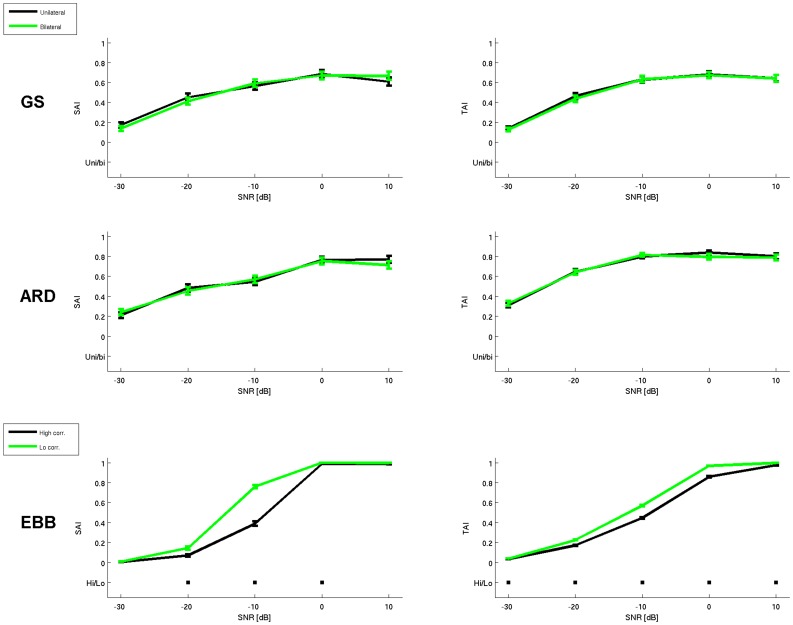
Statistical validation of performances under conditions of high correlation. Upper panel: Plots of ARD and GS performances with highly correlated symmetrical sources in case of inclusion (green) or exclusion of symmetric source priors (black). Lower panel: Differences between EBB performances with high (black) and low (green) correlated sources. The black squares represent significant differences in correspondence of the different SNRs.

In the following, spatial accuracy results for one, two and three sources are separately reported. Fig. 9 provides a descriptive summary of overall performances across search-sizes and SNRs. Fig. 10 and 11 quantify these differences for a fixed search-size of 14 mm. This choice is based on the fact that the G function with *s* = 0.6 has a full width half maximum of two to three mesh vertices. Since the mean distance between vertices is around 5 mm, we considered a search-size of 14 mm as a reasonable trade-off between spatial accuracy and computational constraints. 50 simulations were performed in each condition with different source locations; for each simulation a SAI/TAI test was computed over 100 trials. Fig. 10 and 11 show mean and standard error bars based on the average of these 50 simulation runs. The squares represent significant differences between performances (p<0.05, Bonferroni corrected for number of conditions and SNRs).

#### 1.1 One Source

GS reaches 80% accuracy for SNRs of −10 dB or above when considering the 14 mm search-size (Fig. 9). The spatial accuracy for GS decreases markedly at lower SNRs, but not as abruptly as that of EBB. For the same search-size ARD shows a remarkably robust performance (70–80% of accuracy) even at SNRs as low as −30 dB. At SNR = −20 dB, EBB still shows a localization accuracy of 86% that exceeds not only the localization performance of GS and ARD, but also matches the spatial resolution of the cortical mesh. However, at very low SNRs (−30 dB), EBB accuracy drops to 2%. Fig. 10A (top left panel) quantifies the relative performance of the algorithms Under this condition, ARD performance is significantly better than EBB at −30 dB, whereas the EBB performs significantly better than GS at higher SNRs. We found no significant difference between the performance of the ARD and GS algorithms.

Impressively, MNM maintains a robust performance (70–80% accuracy) for a search-size down to 10 millimeters and a SNR down to −30 dB.

#### 1.2 Two sources

In our simulations for two dipoles, we specifically addressed the question whether correlations between the sources affect the algorithms’ performance. Furthermore, we investigated whether GS and ARD benefit from including symmetric patches to model correlated source priors.

Not surprisingly, when two sources are present instead of one, the localization performance of all algorithms declines. EBB performance deteriorates more rapidly than ARD/GS with decreasing SNR. The plots in the second row of Fig. 10A, show the algorithm’s performance with symmetrically and asymmetrically positioned sources with high (r>0.8) or low (r<0.3) correlation. The curves across all conditions are remarkably similar and show an interaction between algorithm type and SNR with ARD/GS performing more robustly at low SNR (<−10 dB) and EBB showing improved performance at higher SNR (>0 dB).

To our surprise, GS and ARD did not benefit from the inclusion of symmetric priors: highly correlated sources placed on bilateral patches were localized by GS and ARD with comparable accuracy irrespective of the inclusion of bilateral sources priors (see Fig. 11A for a direct comparison). This would also explain the similar performance of the ARD/GS algorithms whether the sources were placed symmetrically or not: in fact, no significant difference between the performance of the ARD and GS algorithms were found in this case either. As expected, in contrast to ARD/GS, the higher correlation between sources does significantly affect the accuracy of the beamformer reconstructions (Fig. 11B). For EBB, pooling across SNRs and taking a search-size of 14 mm, a high *vs.* low correlation performance two sample *t*-test yields a significant difference (*t* = −4.5, N = 1000, p<10^−6^).

In this case MNM performs significantly worse than all other schemes and has reasonable performance only for very large search-sizes and high SNRs.

#### 1.3 Three sources

Performances are similar to the two source case with the EBB performing worse at lower SNR but better at high SNR when compared to ARD/GS. Again, we found no significant difference between the performance of the ARD and GS algorithms. In line with the findings for two sources, the beamformer performance was degraded by correlations between the sources (high *vs.* low correlation performance: two sample *t*-test, search-size 14 mm: *t* = −2.32, N = 250, p<0.05). As in the case of two sources, MNM has the poorest performance, though no further deterioration from 2 sources is detected.

For all algorithms there was no significant decrease in accuracy compared to the two source performance with the exception of EBB at 0 dB and −10 dB (2 *vs.* 3 sources, asymmetrical configuration: *t* = 2, N = 100, p<0.05).

### 2 TAI Results

#### 2.1 Two sources

GS shows a good performance across all the simulations for two and three sources (Fig. 9). Generally, the temporal accuracy is good (70% accuracy) for a search-size between 10 and 15 mm and for SNR levels between +10 and −10 dB. Accuracy decreases at −20 dB and the temporal reconstruction becomes unreliable at −30 dB. ARD maintains at least 70% accuracy at 10 mm for SNRs between 10 and −10 dB.

Fig. 10B quantifies the above for a search-size of 14 mm. The overall picture remains similar to the spatial accuracy results. Nevertheless, some subtle differences are detectable. Firstly, ARD performs consistently better than GS in terms of temporal accuracy. Secondly, the inflection point at which all algorithms have similar performance has increased up to around 0 dB. This highlights the relatively poorer performance of EBB in terms of temporal reconstruction.

#### 2.2 Three sources

The temporal accuracy for three sources mirrors the performance of two. Still, there are significant differences between the algorithms when looking at the relative deterioration due to the increase of sources. While the highest SNRs (10 and 0 dB) do not show any meaningful deterioration in the performance for any algorithm, ARD and EBB, in contrast to GS and MNM, show a decreased performance at −10 dB (2 *vs.* 3 sources: *t* = 2.6, N = 250, p<0.05 for ARD, *t* = 3.2, N = 250, p<0.05 for EBB).

### 3 Free Energy Results

To address the question whether the Bayesian model evidence based on the individual source reconstructions co-varied with our estimates of spatial and temporal accuracy, we used a random effects Bayesian model selection [44] to compare the free energy of solutions for each pair of algorithms over simulations. This results in an exceedance probability or the probability that a particular model is more likely than the other (or any other for more than two models). Generally these results are consistent with the SAI/TAI findings, with high exceedance probability in favour of ARD over GS at low SNR; the difference decreasing with increasing SNR (with SNR>0 dB this approached chance level, 0.5). Similarly, we find that ARD over EBB models are favoured at −30, −20 and −10 dB (p = 0.9999), with model probabilities becoming comparable at around 0 dB. For simulations with high correlation this difference remains marginal at high SNR (10dB), whereas for simulated sources with intrinsically low correlation the exceedance probability in favour of ARD is negligible (i.e. the EBB solution is favoured) We find MNM to be less likely (p<10^−6^) than all the other models for all conditions and SNRs except when compared to the EBB model for low SNR data where the probability of the two models became comparable when source correlation is high.

Moreover, we used Bayesian model selection [44] to pool the evidence over realisations (and conditions) and test whether there was more support for a model using bilateral correlated priors over unilateral ones. The numbers reported here correspond to the expectation of the posterior for the bilateral model. Over all conditions simulated there was no evidence in support of either model (GS: p = 0.47; ARD: p = 0.52). This was true for both the low correlation conditions, where as expected, the addition of bilateral priors had little effect (GS: p = 0.46; ARD: p = 0.51); and also at high correlation, when the underlying distribution was asymmetrical (GS: p = 0.39; ARD: p = 0.44). Even in the case where the sources were symmetrical, the bilateral model was only marginally more likely (GS: p = 0.58; ARD: p = 0.61).

Overall, there was no evidence that the bilateral priors were advantageous. These results confirm that the free energy values provide a useful quantification of the best empirical priors without knowledge of true source locations or time-courses.

## Discussion

By comparing traditional techniques with a Bayesian approach (MNM; EBB) and two Multiple Sparse Priors schemes (ARD and GS), this study complements the existing MEG Bayesian literature focused on classical priors like Equivalent Current Dipole [42], [45], [46] and Minimum Norm [10], [45]. All of these schemes are examples of parametric empirical Bayes. While not Bayesian in the strictest sense [35], Empirical Bayes has been employed in several fields [47] and applied to M/EEG data [12], [45]. Its core difference to traditional Bayes is the concept that the parameters can be estimated from the data. The beamformer prior itself is calculated according to [5] and [36]. This is empirical formulation is exemplified in the EBB scheme in which the data covariance directly determines the prior.

Although, GS and ARD never reach a spatial accuracy comparable to the spatial resolution of the cortical mesh, this can be explained in part by the spatial pattern profile of the covariance components (see MAP equations in Appendix S1). From the results shown in Fig. 3, a cohort of 20 vertices around the center of the patch have comparable (60% or greater) intensity to the central vertex where the dipole is located (*s* = 0.6). A smaller value of the *s* parameter together with an increased number of patches would have probably improved this bound, at the expense of a larger search space for the non-linear optimization. One should also note that we did not simulate patch-like sources but used single dipolar elements as sources. The disparity between the leadfields of single dipoles and the ones of these elements will be greatest when the patches are curved [21], [48]. This could explain the improved performance of EBB over the MSP schemes at high SNR.

Another major finding is that symmetrical correlated priors are not particularly advantageous for GS or ARD from the point of view of spatial and temporal accuracy (SAI and TAI results, *uni vs bi*, Fig. 9).One reason why bilateral patches might have less flexibility is that these priors also imply that the sources in each hemisphere must have approximately the same variance. The use of unilateral priors allows this disparity in variance to be addressed. In practice also it may be that unless the symmetric sources are perfectly correlated the use of a bilateral prior is too restrictive as compared to two unilateral ones.

The evidence that functional networks of neural assemblies can show different correlated hubs within the same hemisphere is constantly growing [49]–[55]. For this reason, it becomes increasingly difficult to construct a priori hypotheses which can cover the whole range of possible functional results. Our results show that such a priori knowledge is not necessary for ARD or GS and indeed one would expect that the removal of these extra (redundant) priors would make the inversion more robust (by decreasing the parameter space of the non-linear search and avoiding possible false positives as in Fig. 6, lower panel). Our results for ARD and GS are consistent with the theoretical proof provided in [4], [36] where an analogous ARD approach was used.

It should be noted that the simulated conditions in this paper were close to ideal: accurate knowledge of the cortical mesh location; the assumed noise model (Gaussian, white) matches exactly the simulated one; sources at patch centers. The robustness of these algorithms under different conditions remains to be investigated.

On that note, our analysis is based on the assumption of stationarity of the sinusoidal basis sets over the time window of interest. Future work might however consider alternative temporal basis functions and so maximize the sensitivity to transient phasic phenomena. We would expect the choice of the temporal basis functions to improve or degrade all algorithms by a similar amount.

In contrast to our initial expectations that the EBB algorithm would be more robust to noise, it showed relatively poor performance at low SNR [56]. At first we thought this could be due to a large number of local maxima produced by the EBB inversion (in which every source has some non-zero value) being penalized by the SAI metric (where maxima outside the search region are punished); increasing stringency of our criteria for a local maximum and taking the top 10%, rather than the top 95% actually degraded the performance even further. Critically, it would seem that the single EBB prior does not give the algorithm the necessary degrees of freedom to explain low SNR data. That is, the global maximum at the source level is determined directly from the sensor level covariance matrix. ReML, employing the single EBB prior, can do nothing but scale this source distribution. If the dominant eigenvalue does not correspond to the true maximum, then the peak current estimate will be at the wrong location. This would explain the performance step in EBB for SNRs higher than 0 dB (see Fig. 10A). In this work we constructed the data dependent priors based on the raw data covariance matrix. Future work might examine the use of priors based on a more compact representation of this matrix prior [57]. Indeed, a number of derivatives of the pure LCMV beamformer exist. For instance, the pseudo-Z beamformer could be implemented under the present framework basically by normalizing the LCMV prior with the noise covariance matrix. However, this would introduce a pseudo-contrast not available in the current implementation of the algorithms based around anatomical priors. Therefore, we settled for keeping focus and consistency, albeit at the expense of suboptimal performance of EBB. MNM outperforms GS and ARD only in the single source case. Under more complex source conditions it performs worse, by our metrics, than the other three schemes. Rather than discounting the MNM algorithm (shown to be rather robust in a number of studies), it should be noted that we have chosen an evaluation scheme (SAI) that focuses on spatial precision, which might be non-optimal for methodologies assuming smooth distributed sources.

ARD and GS employ different approaches to the recursive tuning on hyperparameters: ARD associates one hyperparameter to each source prior, while GS assigns the hyperparameters to source prior sets. A second important difference between ARD and GS is that ARD progressively discards the irrelevant covariance components. In contrast, GS, not only eliminates irrelevant sets of patches, but also generates a new set at each ReML iteration. This process, which alternates pruning and generation of components, is the most versatile of the schemes we have considered. However, ARD and GS did not generally differ in spatial accuracy and ARD, in addition to being simpler, outperformed GS on temporal accuracy. In the future it might be interesting to look at source prior sets provided from different schemes that can be inserted in the GS process, as a sort of *metascheme* which evaluates results generated by different algorithms (e.g. the EBB prior could be part of the library). Moreover, it could be beneficial not to discard the covariance component sets after just one unfavorable ReML choice.

We were encouraged that, having evaluated the performance of the algorithms purely in terms of localization performance, inspection of the Free energy values (which do not depend on explicit knowledge of the solution) would have lead us to the same conclusion. This means that a Bayesian Model Averaging scheme (BMA) [58] can be directly applied to our results to produce a weighted average of the posterior current distributions from the four algorithms. Based on our free energy values this scheme would give larger weight to ARD at low SNR and favor the EBB solution at high SNR (i.e. produce high resolution images when there was sufficient SNR to merit it). Alternatively, by setting the priors in a compatible form, it would be also be possible to produce all possible covariance models in parallel, and weight them using same final ReML scheme (Fig. 1, step 4).

## Supporting Information

Appendix S1 (DOCX)
