# Supplementary material for: Source Reconstruction Accuracy of MEG and EEG Bayesian Inversion Approaches
Source: PLoS One. 2012 Dec 21;7(12):e51985. doi: 10.1371/journal.pone.0051985 (PMC3527408; doi:10.1371/journal.pone.0051985)
Supplement: Appendix S1 — (DOCX) [file pone.0051985.s001.docx]

**Appendix S1:**

**Model inversion and Restricted Maximum Likelihood (ReML)**

ReML is an optimized estimator of variance components derived from the Maximum Likelihood method (MLE). MLE is applied to the data assuming a given statistical model for the estimation of the parameters that maximize the probability of the observed results (given the model, i.e. maximization of the likelihood function). Usually, a Gaussian distribution is assumed for the data with some unknown mean and variance.

The Maximum Likelihood method applied to the estimation of variance components can be extremely computationally demanding with some data sets. In contrast to MLE, the Restricted Maximum Likelihood (ReML) method estimates the model variance components by maximizing the likelihood while taking into account the loss of degrees of freedom resulting from the estimation of fixed effects [[18](#_ENREF_18)]. In our case, this is provided by the spatial and temporal projectors **U** and **T** (see the *preprocessing* section in the main text). This enables the definition of a subspace data matrix with linearly independent columns. The ReML procedure is supplemented with moderately uninformative Gaussian hyperpriors. The iterative maximization of the log-likelihood $ln(\hat{p}(\tilde{\mathbf{B}}))$ is performed by means of a Fisher scoring procedure. In our case the objective function to maximize is the free energy F that constitutes a lower bound to the log-likelihood.

The maximization is usually achieved by means of an Expectation-Maximization procedure [[59](#_ENREF_59)] which alternates between maximizing F with respect to the distribution over the source parameters (E-Step) and the moments of the hyperparameter distribution (M-step) until convergence (the procedure ends when F stops increasing or when only one covariance component corresponding to one patch (ARD, [[4](#_ENREF_4),[14](#_ENREF_14),[60](#_ENREF_60)]) or one patch set (GS, [[17](#_ENREF_17)]) is left).

In practice, the maximization of F is performed in SPM by means of a procedure which is formally equivalent to ReML and features the iteration exclusively on the M-step. The embedding of the E-step into the M step is obtained through the substitution of (3) into (2). In this way, only the final level parameters are left in addition to the random effects. The moments of the hyperparameters are iteratively estimated in the M-step. Upon convergence of the M-step, the parameters of the source distribution are calculated only once in the E-step.

The hyperparameter connected to each patch (or patch set, in the case of GS) is supplied with a weakly informative Gaussian prior distribution *p*(**λ**) = . The a priori moments are the same for each hyperparameter: η = -32 and Π = 1/256; in this way, every scale parameter exp(*λ_i_*) is provided with an extremely large variance and a small expectation value.

The Laplacian fixed form approximation involves the following assumption on the posterior distribution of every *λ_i_*:

A Maximum A Posteriori (MAP) estimate of **λ** = [λ_1_,…,λ_Nc_] is obtained by maximizing *ln*p(**B**,**λ**). Once the mode $\hat{\mathbf{\lambda}}$ is obtained, we set **μ**^λ^ = $\hat{\mathbf{\lambda}}$ (therefore, in the case of ARD, if the conditional mode of the scale parameter $\text{exp}\left(\mu_{^{i}}^{\lambda}\right)$ goes to zero, so does the corresponding variance. Then, the variances of the conditional density and the posterior distribution are matched at this mode using $\Sigma^{\lambda}= -F_{\lambda\lambda}\text{(}\mathbf{\mu}^{\lambda}\text{)}$.

Under these assumptions, the free energy bound on the log-evidence is expressed by F in (11) which is rewritten here for the sake of clarity

$F = -\frac{\nu}{2}tr\text{(}\Sigma\text{(}\mathbf{\mu}^{\lambda}\text{)}^{-1}\mathbf{C}_{\tilde{\mathbf{B}}}\text{)} -\frac{\nu}{2}\ln|\Sigma\text{(}\mathbf{\mu}^{^{\lambda}}\text{)}|- \frac{u\nu}{2}\ln2\pi+ \frac{1}{2}\ln|\text{Σ}^{\lambda}\mathbf{\Pi}\text{|} - \frac{1}{2}\text{(}\mathbf{\mu}^{\lambda}-\mathbf{\eta}\text{)}^{T}\mathbf{\Pi}\text{(}\mathbf{\mu}^{\lambda}-\mathbf{\eta}\text{)}$

**M-Step**

$\begin{matrix} F_{\lambda_{i}} = -\frac{\nu}{2}tr(\mathbf{P}_{i}\text{(}\mathbf{C} - \Sigma\text{(}\mathbf{\mu}^{\lambda}\text{)))} - \Pi_{ii}\text{(}\mu_{i}^{\lambda}- \eta_{i}\text{)} \\ F_{\lambda_{i}\lambda_{j}}=-\frac{\nu}{2}tr(\mathbf{P}_{i} \Sigma\text{(}\mathbf{\mu}^{\lambda}\text{)}\mathbf{P}_{j} \Sigma\text{(}\mathbf{\mu}^{\lambda}\text{)))} - \Pi_{ij} \\ \Delta\mu^{\lambda}=-F_{_{\lambda\lambda}}^{-1}F_{\lambda} \\ \Sigma^{\lambda}= -F_{_{\lambda\lambda}}^{-1} \end{matrix}$

$P_{i} = -\exp\text{(}\lambda_{i}\text{)}\Sigma\text{(}\mathbf{\mu}^{\lambda}\text{)}^{-1}Q_{i}\Sigma\text{(}\mathbf{\mu}^{\lambda}\text{)}^{-1}$ denotes the derivative of the inverse estimated covariance, i.e. the data precision.$F_{\lambda}$and $F_{\lambda\lambda}$are the first derivative and the curvature of F with respect to the hyperparameters.

**E-step** (source parameters, calculated only once)

The E-step calculates the source parameters by means of a Maximum A Posteriori (MAP) inversion.

$$\mathbf{M} = \hat{\mathbf{\Sigma}}^{\varepsilon}(\mathbf{\mu}^{\lambda})\tilde{\mathbf{L}}^{T}\mathbf{\Sigma}(\mathbf{\mu}^{\lambda})^{-1}$$

The first and second moment of the source distribution (i.e. the set of the estimated source time series and their covariance respectively) are given by (Friston et al., 2008b):

$\tilde{\mathbf{S}} = \mathbf{M}\tilde{\mathbf{B}}$ (14)

$$\hat{\Sigma}^{S}= \hat{\Sigma}^{\varepsilon} - \mathbf{M}\tilde{\mathbf{L}}\hat{\Sigma}^{\varepsilon}$$
